# Assessing the Microcirculation With Handheld Vital Microscopy in Critically Ill Neonates and Children: Evolution of the Technique and Its Potential for Critical Care

**DOI:** 10.3389/fped.2019.00273

**Published:** 2019-07-09

**Authors:** Özge Erdem, Can Ince, Dick Tibboel, Jan Willem Kuiper

**Affiliations:** ^1^Intensive Care and Department of Pediatric Surgery, Erasmus University Medical Center – Sophia Children's Hospital, Rotterdam, Netherlands; ^2^Department of Intensive Care, Erasmus University Medical Center, Rotterdam, Netherlands; ^3^Department of Translational Physiology, Amsterdam University Medical Center, Amsterdam, Netherlands

**Keywords:** microcirculation, hemodynamic monitoring, neonates, pediatrics, critical care

## Abstract

Assuring adequate tissue oxygenation in the critically ill, but still developing child is challenging. Conventional hemodynamic monitoring techniques fall short in assessing tissue oxygenation as these are directed at the macrocirculation and indirect surrogates of tissue oxygenation. The introduction of handheld vital microscopy (HVM) has allowed for the direct visualization of the microcirculation and with this has offered insight into tissue oxygenation on a microcirculatory level. Since its introduction, technical improvements have been made to HVM, to both hardware and software, and guidelines have been developed through expert consensus on image assessment and analysis. Using HVM, the microcirculation of the skin, the buccal mucosa, and the sublingual mucosa of healthy and (critically) ill neonates and children have been visualized and investigated. Yet, integration of HVM in hemodynamic monitoring has been limited due to technical shortcomings. Only superficial microcirculatory beds can be visualized, inter-observer and intra-observer variabilities are not accounted for and image analysis happens offline and is semi-automated and time-consuming. More importantly, patients need to be cooperative or fully sedated to prevent pressure and movement artifacts, which is often not the case in children. Despite these shortcomings, observational research with HVM in neonates and children has revealed the following: (1) age-related developmental changes in the microcirculation, (2) loss of hemodynamic coherence, i.e., microcirculatory disturbances in the presence of a normal macrocirculation and, (3) microcirculatory disturbances which were independently associated with increased mortality risk. Although these observations underline the importance of microcirculatory monitoring, several steps have to be taken before integration in the decision process during critical care can happen. These steps include technological innovations to ease the use of HVM in the pediatric age group, measuring additional functional parameters of microvascular blood flow and integrated automated analysis software. As a next step, reference values for microcirculatory parameters need to be established, while also accounting for developmental changes. Finally, studies on microcirculatory guided therapies are necessary to assess whether the integration of microcirculatory monitoring will actually improve patient outcome. Nevertheless, HVM remains a promising, non-invasive tool to help physicians assure tissue oxygenation in the critically ill child.

## Introduction

Hemodynamic monitoring plays a pivotal role in assessing an existing imbalance between oxygen delivery and oxygen consumption and guiding therapy to recover such an imbalance in critically ill neonates and children. Conventional hemodynamic monitoring techniques are aimed at the macrocirculation, i.e., the heart and larger blood vessels, and therefore do not offer insight into oxygen delivery on a cellular level (1–3). A well-functioning macrocirculation alone is no guarantee for adequate oxygen delivery at the cellular level, as it remains unclear whether the microcirculation, comprising smaller arterioles, capillaries, and venules, performs equally well to provide sufficient oxygen delivery.

With the introduction of handheld vital microscopy (HVM) techniques almost 20 years ago, it became possible to assess the microcirculation in a non-invasive manner at the patients' bedside. Microcirculatory research since then has revealed loss of hemodynamic coherence, microcirculatory disturbances in the presence of normal macrocirculatory hemodynamics (4). Loss of hemodynamic coherence can occur either when the recovery of macrocirculatory hemodynamics is insufficient to also recover microcirculatory disturbances or when manipulation of macrocirculatory hemodynamics causes microcirculatory disturbances. In adults with septic shock, Hernandez et al. showed that dobutamine administration improved global hemodynamic parameters, while it failed to improve the sublingual microcirculation and other peripheral perfusion parameters (5). Comparable signs of loss of hemodynamic coherence have also been found in children. In neonates with congenital diaphragmatic hernia, catecholaminergic drugs improved macrocirculatory hemodynamic parameters including mean arterial pressure and heart rate, but could not recruit the microcirculation (6).

These found microcirculatory disturbances have shown to be associated with higher mortality risk (7–9). To illustrate, a study on a mixed group of critically ill adult patients showed the microcirculation to be an independent predictor of ICU mortality, even when corrected for the Acute Physiology, Age, Chronic Health Evaluation (APACHE) II score which includes macrocirculatory parameters (7). Similarly, the microcirculation of pediatric patients with septic shock on the first day could better predict mortality than the Pediatric Risk of Mortality (PRISM) score which also includes macrocirculatory parameters (8). Pediatric patients who underwent therapeutic hypothermia post cardiac arrest but did not survive also showed persistent microcirculatory disturbances, while global hemodynamics did not differ between survivors and non-survivors (9).

Despite the continuous improvement of and the extensive research with HVM, we are still a few steps away from routinely applying microcirculatory monitoring in clinical care. The latest expert consensus on the assessment of the sublingual microcirculation has included important research questions for future observational studies and studies for guiding interventions, but these questions were not specified for neonates and children (10).

This review provides an overview of the different generations of HVM and a summary of studies on the application of HVM in neonates and children. We also discuss important research objectives for future microcirculatory research in these populations and the issues that need to be addressed before clinical application of HVM and integration in current hemodynamic monitoring in neonatal and pediatric critical care is possible.

### Microcirculatory Monitoring

#### Evolution of Handheld Vital Microscopy Techniques

**Figure 1:**
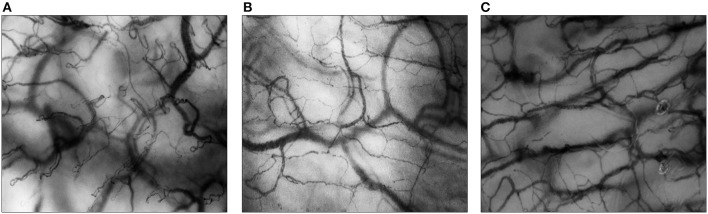
Microcirculatory imaging of different surfaces. All images are assessed with Cytocam-IDF imaging. **(A)** The buccal microcirculation of a neonatal patient; **(B)** the sublingual microcirculation of a pediatric patient; **(C)** the cutaneous microcirculation (upper inner arm) of a preterm neonate.

Over the years, HVM has become an important tool for animal and human research on the microcirculation. Using HVM, microvascular beds of different types of mucosa and solid organ surfaces can be visualized directly up to a depth of ~1 mm, in real-time, and non-invasively at the patient's bedside. In neonates and children, microcirculatory imaging can be acquired from the buccal and sublingual mucosa (11, 12). In neonates, transcutaneous measurements (e.g., upper inner arm, axilla, ear conch, fossa triangularis) are also possible (13). Figure 1 shows examples of images of the buccal, sublingual, and cutaneous microcirculation assessed with HVM.

In the late nineties, Groner et al. introduced the first generation of HVM, the orthogonal polarization spectral (OPS) imaging device (14). The OPS imaging device illuminates tissue through a light source in a handheld probe. While some light is absorbed by hemoglobin, other light is reflected. By disposing of reflected light by the tissue and having scattered (depolarized) light pass through the system, high-contrast images can be acquired of flowing dark red blood cells (RBC) against a light gray background. In 2007, Goedhart et al. introduced the sidestream dark field (SDF) imaging device, a comparable handheld probe with an integrated pulsating green light source (15). The reflected green light is lead through the center of the probe with a magnifying lens system up to a computer to create images with higher contrast and better quality than that of its predecessor. Finally, in 2015 Aykut et al. introduced the third and latest generation microscope, the incident dark field illumination (IDF) imaging device (16). The device has a lightweight pen-like probe incorporated with IDF and higher resolution lenses. The lenses project images on to a computer with a better density sensor and improved control of illumination and image acquisition. These alterations result in a larger field of view and larger image size with higher resolution than those of its predecessors (17–20). The IDF imaging device provides images of higher quality and accuracy and therefore shows higher vessel densities (17). More details on the technical aspects of the currently available microscopes can be found in the review by Massey et al. (20).

#### Image Acquisition and Analysis

In 2007, De Backer et al. published the first expert consensus paper addressing the requirements for optimal image acquisition and the different parameters necessary for interpretation (21). More recently, Ince et al. published an updated consensus paper specifically discussing the assessment of the sublingual microcirculation in critically ill (adult) patients (10). Currently, there are no special considerations for assessment in pediatric and neonatal patients. Only a single manual for the assessment of microcirculatory imaging has been published, describing measurements of the cutaneous microcirculation in (pre)term neonates (22). Extensive training for both image acquisition and analysis by experienced users is necessary to assure adequate quality of images and reliability of results. Before image acquisition, a clean protective cap is placed on the probe. The surface area is cleaned from any saliva with gauze, if applicable, as excessive saliva can reduce the visibility of vessels and should therefore be removed wherever possible. For transcutaneous measurements, gel, oil or saline is applied on the tip of the probe. The probe is gently placed on the surface area. The focus is adjusted to the degree that single RBC's are visible in the capillaries. Artifacts caused by excessive saliva, air bubbles or pressure should be avoided. For a minimum of 4 s each, motion-free measurements are made of at least three different areas.

After image acquisition image analysis is possible through real-time visual evaluation, offline manual analysis, and offline semi-automated analysis. Massey et al. developed an image quality score to ensure that only imaging of sufficient quality is considered for analysis (23). The three clips with the best quality are selected for image analysis of a single time point. Image analysis provides several quantitative and qualitative parameters for microcirculatory function as described elsewhere and summarized below (10). The average of the three selected clips (of three different spots) is calculated to assess the value of a single time point.

**Figure 2:**
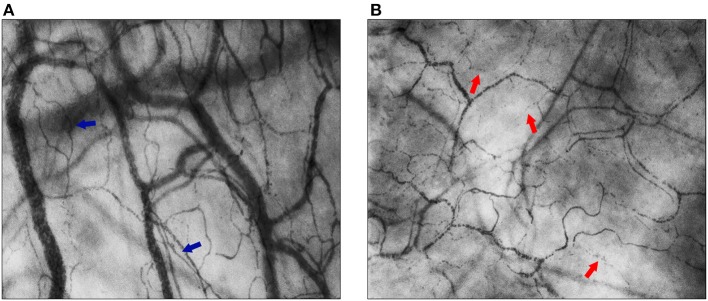
Flow quality of the sublingual microcirculation. In both images, the sublingual microcirculation of neonatal patients is visualized, assessed with Cytocam-IDF imaging. **(A)** Blue arrows point toward capillaries with normal flow (MFI = 3); **(B)** red arrows point toward capillaries with intermittent flow (MFI = 1).

Total vessel density (TVD, mm/mm^2^) quantifies the total vessel area visible in the frame. Before the semi-automated assessment of vessel density was possible, the Backer score (n/mm) was calculated as an estimation of TVD (24). Proportion of perfused vessels (PPV, %) gives the number of perfused vessels per total number of visible vessels in the frame. Perfused vessel density (PVD, mm/mm^2^), in former studies often referred to as functional vessel density (FVD, cm/cm^2^) or functional capillary density (FCD, cm/cm^2^) for small vessels, describes the functional vessel area visible in the frame, through the multiplication of TVD and PPV. TVD and PVD can be used to assess alterations in diffusive properties of the microcirculation as these parameters are surrogates for the distance oxygen has to cover from capillary to cell. As for flow properties, quality, heterogeneity, and velocity of flow can be determined. Microcirculatory flow index (MFI) is a semi-qualitative score to describe the quality of flow through the microvascular vessels. Figure 2 shows two examples of continuous flow and intermittent flow, respectively. The MFI can be assessed in real-time (25, 26). To account for variability between measured areas, the heterogeneity index (HI) can be calculated for PPV and MFI through the equation *(highest value–lowest value)/mean value*. HI can help determine the presence of abnormalities in flow distribution. The increase of heterogeneity of flow can be a sign of functional shunting. To assess the velocity of flow, RBC velocities can be measured using space-time diagrams (STD). Recently, Uz et al. developed a method to also count leukocytes and assess leukocyte velocity in microcirculatory imaging using STD (27). Fabian-Jessing et al. also developed a method for assessing and quantifying leukocyte rolling and adherence (28). Finally, to assess early markers of vascular wall damage endothelial glycocalyx layer dimensions can be estimated through the width of flowing RBC's (29).

Currently available analysis software is semi-automated and therefore time-consuming. Automated analysis software has been developed, but these are not yet validated for use in clinical practice (30–34). Currently, online automated analysis software is being developed to obtain the above mentioned and additional parameters more easily and rapidly.

#### Limitations

Despite technological advancements and the extensive application of HVM in research, there are still several limitations to their use. The microcirculation can only be visualized if the epithelial layer of the area of interest is thin. Also, pressure and movement artifacts are not rare. Although its use is validated in children and neonates, the acquisition is complicated as children need to be sufficiently sedated or fully cooperative to attain good quality imaging (13, 35, 36). Gonzalez et al. performed an observational study in the pediatric ICU, where sublingual microcirculatory monitoring was only possible in 17% of the admitted patients in the study period (37). These patients were sedated and/or more severely ill. As for microcirculatory measurements of the skin, these are performed in the first few weeks of life in both preterm and term neonates. Thickening of skin with age and the presence of hair interferes with optimal visualization of the cutaneous microcirculation. There is no exact limit for age for the assessment of the cutaneous microcirculation, as this has not been investigated. Van Elteren et al. has looked into the difference in transcutaneous microcirculation of preterm and term neonates and stated that depth of focus depended on postnatal age rather than gestational age (22). In both groups, it was more difficult to assess images on day 28. For image analysis, only offline semi-automated analysis software is currently available and existing automated software programs are not yet validated. Therefore actual bedside evaluation of the microcirculation is not possible, although MFI could be assessed at the patient's bedside through eyeballing (25, 26, 38). Because the semi-automated analysis is currently the gold standard for image analysis, inter-observer, and intra-observer variabilities need to be addressed. Lima et al. found a moderate agreement of assessing microcirculatory disturbances between real-time assessment and offline analysis (39). As for the offline analysis of SDF imaging with semi-automated software, van den Berg et al. found that vessel density of the buccal microcirculation was highly reproducible, while vessel density of the cutaneous microcirculation was not and showed high variabilities (35). It remains unclear how great inter-observer and intra-observer variabilities are for the sublingual microcirculation and if these variabilities differ with the use of different generations of HVM.

Currently, reference values for microcirculatory parameters for neonates and children are lacking. Only two small observational studies have been performed in healthy term neonates and no studies have been performed in healthy children (35, 36). There is insufficient knowledge of intra-individual heterogeneity, the natural fluctuation of measured variables and the correlation between different microvascular beds under healthy and pathological conditions (40). It is also important to address developmental changes through aging and the differences in values of microcirculatory variables derived from the different techniques and from different microvascular beds (17). This creates a challenge for comparing research findings, especially for comparing measurements or using reference values made with newer, more sensitive techniques. Results derived from older techniques are not obsolete as the vast majority of these studies evaluate change over time, change after intervention, or differences between groups. However, all studies conducted in children (as described in the following paragraph) have been observational.

### Microcirculation Research in Neonates and Children

Since its introduction, HVM has also found its way in neonatal and pediatric research, although only in rather small observational studies. Table 1 gives a summary of the microcirculatory studies performed with HVM in preterm and term neonates and Table 2 gives a summary of the studies performed in pediatric patients. Genzel-Boroviczény et al. were the first to use OPS imaging on the cutaneous microcirculation of term and preterm neonates (13). The skin of the inner upper arm was the most feasible area; here the skin is thin, without hair and measurements are less prone to movement artifacts caused by breathing. Later on, both SDF imaging and IDF imaging were used on the cutaneous and buccal microcirculation (17, 35, 36). While buccal FVD values were highly reproducible, cutaneous FVD values were not. As for pediatric patients, either the buccal or the sublingual microcirculation have been areas of interest as apparent from Table 2.

**Table 1:** Summary of findings: microcirculatory studies performed in neonates.

**Reference**	**HVM**	**Study population**	***n***	**Area of interest**	**Findings**
Genzel-Boroviczeny et al. (13)	OPS	Healthy preterm vs. term neonates	28/9	Cutaneous (upper inner arm)	Application OPS imaging; groups did not differ; RBC velocity increased from day 1 to 5 in preterm neonates alongside decrease of Ht
Genzel-Boroviczeny et al. (41)	OPS	Anemic preterm neonates receiving blood transfusion	13	Cutaneous (upper inner arm)	FVD increased after blood transfusion; other microcirculatory or macrocirculatory parameters were unaltered
Kroth et al. (42)	OPS	Healthy preterm neonates	25	Cutaneous (upper inner arm)	FVD decreased from week 1 to 4 and was correlated with Hb and incubator temperatures; VD and RBC velocities did not change over time
Weidlich et al. (43)	OPS	Preterm neonates: proven infection vs. suspected but unproven infection	17/9	Cutaneous (upper inner arm)	FVD varied widely, infection group showed 10% decline 5 days before AB compared to controls (intra-individual differences)
Top et al. (11)	OPS	Term neonates with severe respiratory failure: VA ECMO vs. controls	14/10	Buccal mucosa	FVD of ECMO patients was lower before start ECMO than of controls; FVD improved after ECMO
Hiedl et al. (44)	SDF	Preterm neonates: significant PDA vs. non-significant PDA	13/12	Cutaneous (upper inner arm)	Group with significant PDA showed lower FVD and higher number of small vessels; after treatment groups did not differ
Top et al. (45)	OPS	Healthy term neonates vs. 1 to 6 month olds vs. 3 year olds	22/19/4	Buccal mucosa	FVD was highest in first week of life; after first week no correlation between FVD and age
Ergenekon et al. (46)	SDF	Neonates with polycythemia requiring partial exchange transfusion	15	Cutaneous (axilla)	After transfusion MFI and number of vessels with hyperdynamic flow increased from baseline values
Top et al. (47)	OPS	Term neonates with severe respiratory failure: VA ECMO vs. controls	21/7	Buccal mucosa	FVD is preserved after start ECMO, while FVD deteriorated in ventilated controls
Alba-Alejandre et al. (48)	OPS	Term neonates: mild/moderate infection vs. controls	16/31	Cutaneous (ear conch)	Infection group showed lower PPV with continuous flow than controls
Schwepcke et al. (49)	SDF	Preterm neonates: postnatal hypertension vs. controls	10/11	Cutaneous (upper inner arm)	Preterm neonates with hypotension showed higher FVD in the first 6 h after birth; at 12 h after birth both blood pressure and FVD did not differ between groups
Tytgat et al. (12)	SDF	Neonates undergoing laparoscopic surgery for hypertrophic pyloric stenosis	12	Buccal and sublingual mucosa	Buccal FVD did not differ before and after surgery. Sublingual blood vessel diameters increased during CO_2_ insufflation and decreased after CO_2_ exsufflation
Ergenekon et al. (50)	SDF	Term neonates with HIE: TH vs. controls	7/7	Cutaneous (axilla)	Patients showed lower MFI and more vessels with sluggish flow than controls. After TH parameters recovered to values of controls
Buijs et al. (6)	SDF	Term neonates with CDH: catecholamines vs. controls	28/28	Buccal mucosa	Catecholamines improved the macrocirculation, but did not alter the microcirculation; impaired microcirculation was predictive of outcome
Van den Berg et al. (35)	SDF	Healthy term neonates	28	Cutaneous (upper inner arm)/buccal mucosa	Application SDF imaging; reproducibility of buccal PVD with SDF imaging was confirmed, cutaneous PVD showed poor reproducibility
Van Elteren et al. (17)	SDF/IDF	Healthy preterm neonates	20	Cutaneous (upper inner arm)	IDF imaging showed higher TVD and lower PPV values than SDF imaging because of higher image quality
Van Elteren et al. (51)	IDF	Healthy preterm vs. term neonates	60/33	Cutaneous (upper inner arm)	TVD decreased in first month of life in both groups; TVD was higher in preterm than in term neonates
Gassmann et al. (52)	IDF	Healthy term neonates: born at high altitude vs. born at sea level	53/33	Cutaneous (upper inner arm)	TVD was higher in neonates born at high altitude (lower SpO_2_ levels) than in neonates born at sea level
Wright et al. (36)	SDF	Healthy term neonates	42	Cutaneous (ear conch)	Application SDF imaging; reporting of reference values for microcirculatory parameters for ear conch
Kulali et al. (53)	SDF	Healthy term neonates: vaginal delivery vs. cesarean section	12/25	Cutaneous (axilla)	Vaginal delivery group showed more vessels with hyperdynamic flow than cesarean section group; other parameters did not differ between groups
Puchwein-Schwepcke et al. (54)	SDF	Term neonates: infection treated with antibiotics vs. controls	13/95	Cutaneous (ear conch)	Infection group showed lower FVD and higher proportion of hyperdynamic flow than control group; hyperdynamic flow was associated with 5-fold increased risk for infection
Puchwein-Schwepcke et al. (55)	SDF	Preterm neonates with extreme LBW: hypercapnia vs. controls (sub-analysis RCT)	6/6	Cutaneous (upper inner arm)	Hypercapnia group showed lower FVD and relatively fewer small vessels than controls

BP, blood pressure; CDH, congenital diaphragmatic hernia; ECMO, extracorporeal membrane oxygenation; etCO_2_, end tidal carbon dioxide; FVD, functional vascular density; GA, gestational age; Hb, hemoglobin; HIE, hypoxic ischemic encephalopathy; HR, heart rate; Ht, hematocrit; HVM, handheld vital microscopy; IDF, incident dark field illumination; LBW, low birth weight; OPS, orthogonal polarization spectral; PDA, persistent ductus arteriosus; PPV, perfused vessel density (%); RBC, red blood cell; SDF, sidestream dark field; TH, therapeutic hypothermia; TVD, total vessel density; VA ECMO, veno-arterial extracorporeal membrane oxygenation; VD, vessel diameter.

**Table 2:** Summary of findings: microcirculatory studies performed in pediatric patients.

**Reference**	**HVM**	**Study population**	**n**	**Area of interest**	**Findings**
Top (56)	OPS	Septic shock	1	Buccal mucosa	Sepsis therapy recovered macrocirculation, while microcirculation was still compromised
Top et al. (8)	OPS	Septic shock: survivors vs. non-survivors	15/3	Buccal mucosa	FVD improved on day 2 in survivors, while FVD was lower and did not change in non-survivors; persistent microcirculatory disturbances were prognostic for mortality
Top et al. (57)	OPS	Hypoxemic respiratory failure: iNO therapy	8	Buccal mucosa	iNO therapy improved FVD
Paize et al. (58)	SDF	Meningococcal disease vs. controls	20/40	Sublingual mucosa	Microcirculatory parameters were lower in meningococcal disease than in controls but recovered when patients clinically recovered
Buijs (59)	SDF	Respiratory failure: VA ECMO vs. VV ECMO	31/17	Buccal mucosa	Groups did not differ; PPV and MFI were impaired prior to start ECMO, improved one day after start ECMO and recovered in both groups
Buijs et al. (9)	SDF	Post cardiac arrest patients during TH vs. controls	22/20	Buccal mucosa	All microcirculatory parameters were impaired during TH; severe impairment at start TH was associated with mortality; microcirculatory parameters improved rapidly after TH
Nussbaum et al. (60)	SDF	Diabetes patients vs. controls	14/14	Sublingual mucosa	Glycocalyx thickness was reduced in diabetes patients compared to controls and inversely correlated with blood glucose levels; diabetes patients showed more large vessels than small vessels than controls
Nussbaum et al. (61)	SDF	Cardiac surgery vs. cardiac catheterization vs. non-cardiac surgery controls	40/6/9	Cutaneous (fossa triangularis ear)	Glycocalyx thickness was reduced after cardiac surgery and returned to baseline values after 1 week; MFI and PVD also declined and returned to baseline values after 24 h
Schinagl et al. (62)	SDF	Anemic children receiving RBC transfusion vs. controls	19/18	Buccal mucosa	Anemic children showed lower TVD lower and higher RBC velocity than controls; after transfusion, Hb and TVD increased and RBC velocity decreased; TVD and RBC velocity did not reach levels of controls
Scolletta et al. (63)	SDF	Cardiac surgery: cyanotic vs. a-cyanotic heart defects	7/17	Sublingual mucosa	Microcirculatory parameters did not change over time and were not correlated to macrocirculation in both groups; cyanotic children showed different time trends for PPV and TVD than a-cyanotic children
Gonzalez et al. (37)	IDF	Admission pediatric ICU	105	Sublingual mucosa	Microcirculatory assessment only possible in 17%, mostly intubated and the more severely ill patients; microcirculatory parameters were moderately correlated with BP, CVP, and lactate
Riedijk and Milstein (64)	IDF	Procedural sedation with propofol	7	Sublingual mucosa	Propofol induction induced a decline of BP and an increase of TVD and PVD

*BP, blood pressure; CVP, central venous pressure; FVD, functional vascular density; Hb, hemoglobin; HVM, handheld vital microscopy; IDF, incident dark field illumination; iNO, inhaled nitric oxide; MFI, microcirculatory flow index; OPS, orthogonal polarization spectral; PPV, perfused vessel density (%); PVD, perfused vessel density; RBC, red blood cell; SDF, sidestream dark field; TH, therapeutic hypothermia; TVD, total vessel density; VA ECMO, veno-arterial extracorporeal membrane; VV ECMO, veno-venous extracorporeal membrane*.

Microcirculatory imaging has been used to assess physiological changes in the microcirculation, in particular developmental changes. Studies with all three generations of HVM in term and preterm neonates showed that the FVD of both the cutaneous and buccal microcirculation and the TVD of the cutaneous microcirculation decreased in the first 4 weeks of life (17, 42, 45, 51). Along the same lines, studies showed that preterm neonates had higher TVD values than term neonates. The parameters of the two microcirculatory beds did not correlate (17). Gassmann et al. observed term neonates born at high altitude with IDF imaging and found higher TVD in the cutaneous microcirculation of these neonates than of those born at sea level (52). Schwepcke et al. investigated the cutaneous microcirculation of both hypotensive as normotensive preterm neonates with OPS imaging and found higher FVD values in the first group (49). Twelve hours after birth these differences had disappeared.

HVM also offers the opportunity to examine how the microcirculation reacts under different circumstances including hypercapnia and administration of inhaled nitric oxide. Puchwein-Schwepcke et al. looked at the effect of hypercapnia on the cutaneous microcirculation of preterm neonates with extremely low birth weight with OPS imaging, as a sub analysis of a RCT (55). High PCO_2_ levels affected the microcirculation by reducing FVD and causing a shift to more functional large vessels than small vessels. Top et al. investigated the effect of inhaled nitric oxide on the buccal microcirculation in children with hypoxemic respiratory failure with OPS imaging. FVD was found to increase with inhaled nitric oxide (57).

Others have used HVM to assess pathophysiological changes in the microcirculation during disease. To illustrate, microcirculatory disturbances have been found in patients with an infection and/or sepsis, with persistently declined FVD and impaired microcirculatory blood flow. Weidlich et al. demonstrated with OPS imaging that FVD declined in preterm neonates with an infection over several days, before clinical suspicion occurred (43). Puchwein-Schwepcke et al. found comparable results in term neonates with SDF imaging on the upper ear conch, as patients with an infection treated with antibiotics showed lower FVD and, in addition, a higher proportion of hyperdynamic flow compared to controls without an infection (54). In contrast, Alba-Alejandre observed different microcirculatory alterations in both the ear conch and the upper arm of term neonates with a mild to moderate infection (48). These patients showed less perfused vessels with continuous flow than patients without an infection, while FVD did not differ between the two groups. Top et al. looked at the buccal microcirculation in 18 children with septic shock with OPS imaging (8). FVD was lower in non-survivors than in survivors. Also, in survivors FVD improved on the second day, while FVD did not change in non-survivors. These findings demonstrated that persistent microcirculatory disturbances irrespective of macrocirculatory hemodynamics could be a sign of worse outcome. Similarly, Paize et al. investigated the sublingual microcirculation of 20 children with severe meningococcal disease with SDF imaging. They found all microcirculatory parameters to be lower in these children than in healthy controls (58). Parallel to clinical recovery, microcirculatory parameters restored to values comparable to those of controls.

HVM can also be applied to assess the effect of different therapies on the microcirculation and the predictive value of persistent microcirculatory impairment. Buijs et al. looked at the effect of therapeutic hypothermia (TH) on the buccal microcirculation in children after cardiac arrest with SDF imaging (9). During TH the microcirculation was impaired, while after TH the microcirculation improved rapidly. Severely altered microcirculation at the start of TH was also associated with mortality. Similar findings were shown in the study with SDF imaging on the effect of TH on the cutaneous microcirculation in neonates with hypoxic-ischemic encephalopathy (50). Buijs et al. monitored the buccal microcirculation of term neonates with congenital diaphragmatic hernia receiving catecholaminergic drugs with SDF imaging (6). Dopamine, norepinephrine, and epinephrine improved macrocirculatory hemodynamics but did not alter microcirculatory function. Impaired microcirculation despite therapeutic efforts was predictive of poor outcome, irrespective of macrocirculatory hemodynamics.

Genzel-Boroviczeny et al. demonstrated the effects of blood transfusion on the cutaneous microcirculation of anemic preterm neonates with OPS imaging (41). After a blood transfusion, FVD increased, while systemic hemodynamics were unaltered. Along the same lines, Schinagl et al. looked at the effect of RBC transfusion on the buccal microcirculation of anemic children with SDF imaging (62). Anemic children showed lower TVD values and higher RBC velocities than controls. After a transfusion, TVD increased and RBC velocities decreased, although still not to values of controls. TVD was highly correlated to hemoglobin levels. It is possible RBC velocities increase to compensate for the decreased oxygen delivering capacity of RBC with anemia. As blood viscosity decrease and arteries dilate, the peripheral resistance is lowered, probably the reason for RBC velocities to increase (65). This would explain why RBC velocities decrease after a transfusion, as these hemodynamic compensating mechanisms are no longer necessary when the oxygen delivering capacity has improved.

Following research findings in adults, the effect of cardiopulmonary bypass on the microcirculation has also been assessed in children. Nussbaum et al. looked at the effect of cardiac surgery on cardiopulmonary bypass on the cutaneous microcirculation with SDF imaging (61). After cardiac surgery, glycocalyx thickness was reduced but recovered to baseline values after 1 week. MFI and PVD also declined postoperatively. In a similar population, Scolletta et al. looked at the sublingual microcirculation, also comparing cyanotic and a-cyanotic heart defects with SDF imaging (63). The sublingual microcirculation was not altered over time, in contrast to previous findings in cutaneous microcirculation. Cyanotic heart defects, however, did seem to affect TVD and PPV. Top et al. attempted to assess the effect of extracorporeal membrane oxygenation (ECMO), another type of cardiopulmonary bypass applied in critical care. They looked into the buccal microcirculation of neonates with severe respiratory failure treated with veno-arterial ECMO with OPS imaging (11). Before the start of ECMO treatment, FVD values were lower than controls and after ECMO treatment FVD values increased. In a similar study population, Top et al. showed that FVD is preserved after starting ECMO treatment, while FVD deteriorated in neonates who were only ventilated (47).

### Future Perspectives

Research with HVM has revealed the added value of microcirculatory monitoring as part of hemodynamic monitoring to determine if oxygen delivery is sufficient to meet metabolic demands, as adequate microcirculatory blood supply is one of the prerequisites for meeting metabolic demands. However, clinical application is not possible before technical improvements have been made and further research has been conducted on microcirculatory guided therapy. This is especially vital for children as most of the research has been conducted in human adults and only rather small observational studies have been performed in children. In this final part, we have summarized important considerations from the previously mentioned expert consensus with special focus on those which are important for neonatal and pediatric patients (10).

#### Technological Innovations

One of the biggest challenges of microcirculatory monitoring in children is acquiring imaging of sufficient quality due to movement and pressure artifacts and lack of cooperation. Currently, HVM measurements are limited to deeply sedated and critically ill patients. Technological innovations are necessary to ease the use in children and thus to have full access to the potential of HVM. Hardware improvements should include pressure recognition and quantification, artifact recognition and warning signaling, and the possibility to perform single spot measurements over a longer period of time, e.g., through hands-free measurements. New software should be integrated with RBC velocity measurements throughout the entire frame, automated assessment of MFI, automated image stabilization and quality assessment and, most importantly, automated analysis software. Real-time monitoring will only be possible if automated analysis software is integrated in the software of the device. Finally, additional parameters are necessary to fully assess microcirculatory function. As MFI is only a qualitative measure for the convective capacity (i.e., blood flow) of the microcirculation, a quantitative measure is needed to assess the actual oxygen-carrying capacity of the microcirculation. This will be possible through the measurement of capillary hematocrit, as proposed by Ince et al.: tube hematocrit (the hematocrit of capillary blood at a single moment in time) and discharge hematocrit (the hematocrit flowing through capillaries per unit of time) (10).

#### Future Research Objectives

In order to study microcirculatory disturbances and attempt to correct these disturbances, first reference and critical values have to be established. For this research objective, we also need to account for differences between different microcirculatory beds and between different generations of HVM and for developmental changes in the microcirculation after birth and through childhood. Also, expert consensus for assessment of the buccal and cutaneous microcirculation in neonates and children would be preferable as no such guidelines exist.

After reference values are established research could take steps toward microcirculatory guided therapy. We need to assess how different microcirculatory beds correlate to one another under pathological conditions. For example, in adult septic patients Edul et al. demonstrated the dissociation between the sublingual and intestinal microcirculation in response to fluid resuscitation (66). While responsiveness of the sublingual microcirculation seemed to be dependent on cardiac output, the intestinal microcirculation responded irrespective of systemic hemodynamics. It cannot be excluded that organ-specific reactivity also plays a role. Therefore, under a variety of clinical conditions, such as laparotomy and thoracotomy, individual organs should be measured alongside the sublingual microcirculation and compared with the latter. Also, we have to establish when loss of hemodynamic coherence occurs in critically ill children as to account for differences between macrocirculatory and microcirculatory disturbances that need to be recovered. Then, it has to be clear when recovery of macrocirculatory hemodynamics is sufficient to recover microcirculatory disturbances and when we need to take additional steps to recover the microcirculation. To illustrate, Dubin et al. demonstrated in adult septic shock patients that while norepinephrine improved macrocirculatory hemodynamics, it did not alter sublingual microcirculatory parameters (67). Buijs et al. made similar observations in neonates with congenital diaphragmatic hernia (6). Holmgaard et al. showed that different levels of mean arterial pressure did not alter the sublingual microcirculation in adults during cardiopulmonary bypass (68).

The next step would be to assess which therapeutic strategies could recover microcirculatory disturbances in children. In adult research few studies have already looked into therapeutic strategies. In septic patients, Dubin et al. also found that microcirculatory monitoring could help distinguish which type of fluid, crystalloid or colloid, would be more effective in recovering microcirculatory blood flow during early goal-directed therapy (69). Spronk et al. performed a small observational study in adult septic shock patients and found that infusion of nitroglycerin might help resolve microcirculatory disturbances as MFI improved (70). Following this finding, Boerma et al. performed an RCT in a similar population, but here infusion of nitroglycerin was unsuccessful in the recruitment of the sublingual microcirculation (71). For therapeutic strategies to be effective, target ranges need to be established for relevant microcirculatory parameters. Pranskunas et al. underlined this by demonstrating that microcirculatory monitoring could help assess which (adult) patients were eligible for fluid therapy (72). Patients with MFI <2.6 were fluid responsive, as MFI increased after fluid resuscitation, while in patients with MFI > 2.6 MFI was unaltered. Van der Voort et al. tested an actual therapeutic strategy incorporated with a microcirculatory target in a pilot study with adults with septic shock (73). Recruitment of the microcirculation in this resuscitation strategy did not resolve organ dysfunction quicker than standard therapy, although the study itself had some flaws in its design. However, this study shows a potential way of looking into whether actively recovering microcirculatory disturbances could help improve patient outcome. Finally, one should also take intra-individual variability in therapy response into account when looking at the effectiveness of therapeutic strategies.

Although some observational studies have pointed toward the existing association between microcirculatory disturbances and outcome, strong evidence for the predictive value of the microcirculation is lacking for children. It has yet to be established that recovery of microcirculatory disturbances will indeed improve patient outcome since there are only two small negative trials, of which one also failed to recover microcirculatory disturbances (71, 73). Based on these trials in adults, there is currently no evidence to support the clinical use of microcirculatory monitoring with HVM in children. However, there might be in the future, since larger trials to evaluate the microcirculation, similar to for example trials on high-frequency oscillatory ventilation or inhaled nitric oxide for acute respiratory distress syndrome, have not yet been carried out (74, 75). A good example to follow would be the study on lactate-guided therapy in critically ill adults performed by Jansen et al. (76). To perform similar trials with microcirculatory monitoring, international research collaborations are necessary as single centers will have insufficient numbers of patients to prove any effect of therapeutic interventions on the microcirculation of children. Nevertheless, we should also be realistic regarding the number of patients and consider the feasibility of such trials. To illustrate, to prove the effect of ECMO treatment in pediatric acute respiratory distress syndrome in an RCT, one would need more than 1,000 patients per treatment arm (77, 78).

## Summary

Integrating microcirculatory monitoring in routine hemodynamic monitoring may be important for the assurance of adequate tissue oxygenation as it offers additional insight into oxygen delivery. HVM is a promising tool for assessing otherwise unnoticed disturbed oxygen delivery on a microcirculatory level. Before application in neonatal and pediatric critical care is possible, however, technological advancements and extensive research on microcirculatory guided therapy are necessary.

## Author Contributions

ÖE and JK wrote the draft of the manuscript. All authors contributed to manuscript revision, read, and approved the submitted version.

### Conflict of Interest Statement

CI has developed SDF imaging and is listed as an inventor on related patents commercialized by MicroVision Medical under a license from the Academic Medical Center in Amsterdam, the Netherlands. He has been a consultant for MicroVision Medical in the past, but has not been involved with this company for over 5 years now and holds no shares. Braedius Medical, a company owned by a relative of CI, has developed and designed a handheld microscope called CytoCam-IDF imaging. CI has no financial relationship with Braedius Medical of any sort, i.e., never owned shares, or received consultancy or speaker fees from Braedius Medical. The remaining authors declare that the research was conducted in the absence of any commercial or financial relationships that could be construed as a potential conflict of interest.

## Abbreviations

APACHE II score, acute physiology, age: chronic health evaluation II score
ECMO: extracorporeal membrane oxygenation
FCD: functional capillary density
FVD: functional vessel density
HI: heterogeneity index
HVM: handheld vital microscopy
MFI: microcirculatory flow index
OPS: orthogonal polarization spectral
PPV: proportion of perfused vessels
PRISM score: pediatric risk of mortality score
PVD: perfused vessel density
RBC: red blood cell
SDF: sidestream dark field
STD: space-time diagram
TH: therapeutic hypothermia
IDF: incident dark field illumination
TVD: total vessel density.
