# Corrigendum: Assessing the Microcirculation With Handheld Vital Microscopy in Critically lll Neonates and Children: Evolution of the Technique and Its Potential for Critical Care

**DOI:** 10.3389/fped.2019.00346

**Published:** 2019-08-21

**Authors:** Özge Erdem, Can Ince, Dick Tibboel, Jan Willem Kuiper

**Affiliations:** ^1^Intensive Care and Department of Pediatric Surgery, Erasmus University Medical Center – Sophia Children's Hospital, Rotterdam, Netherlands; ^2^Department of Intensive Care, Erasmus University Medical Center, Rotterdam, Netherlands; ^3^Department of Translational Physiology, Amsterdam University Medical Center, Amsterdam, Netherlands

**Keywords:** microcirculation, hemodynamic monitoring, neonates, pediatrics, critical care

In the original article, there was a mistake in Table 1 as published. In rows 6 to 10, the wrong references were listed for the findings summarized in these rows. Two additional references with findings were missing in the table due to this error. The corrected Table 1 appears below.

**Table 1:** Summary of findings: microcirculatory studies performed in neonates.

**Reference**	**HVM**	**Study population**	***n***	**Area of interest**	**Findings**
Genzel-Boroviczeny et al. (13)	OPS	Healthy preterm vs. term neonates	28/9	Cutaneous (upper inner arm)	Application OPS imaging; groups did not differ; RBC velocity increased from day 1 to 5 in preterm neonates alongside decrease of Ht
Genzel-Boroviczeny et al. (41)	OPS	Anemic preterm neonates receiving blood transfusion	13	Cutaneous (upper inner arm)	FVD increased after blood transfusion; other microcirculatory or macrocirculatory parameters were unaltered
Kroth et al. (42)	OPS	Healthy preterm neonates	25	Cutaneous (upper inner arm)	FVD decreased from week 1 to 4 and was correlated with Hb and incubator temperatures; VD and RBC velocities did not change over time
Weidlich et al. (43)	OPS	Preterm neonates: proven infection vs. suspected but unproven infection	17/9	Cutaneous (upper inner arm)	FVD varied widely, infection group showed 10% decline 5 days before AB compared to controls (intra-individual differences)
Top et al. (11)	OPS	Term neonates with severe respiratory failure: VA ECMO vs. controls	14/10	Buccal mucosa	FVD of ECMO patients was lower before start ECMO than of controls; FVD improved after ECMO
Hiedl et al. (44)	SDF	Preterm neonates: significant PDA vs. non-significant PDA	13/12	Cutaneous (upper inner arm)	Group with significant PDA showed lower FVD and higher number of small vessels; after treatment groups did not differ
Top et al. (45)	OPS	Healthy term neonates vs. 1 to 6 month olds vs. 3 year olds	22/19/4	Buccal mucosa	FVD was highest in first week of life; after first week no correlation between FVD and age
Ergenekon et al. (46)	SDF	Neonates with polycythemia requiring partial exchange transfusion	15	Cutaneous (axilla)	After transfusion MFI and number of vessels with hyperdynamic flow increased from baseline values
Top et al. (47)	OPS	Term neonates with severe respiratory failure: VA ECMO vs. controls	21/7	Buccal mucosa	FVD is preserved after start ECMO, while FVD deteriorated in ventilated controls
Alba-Alejandre et al. (48)	OPS	Term neonates: mild/moderate infection vs. controls	16/31	Cutaneous (ear conch)	Infection group showed lower PPV with continuous flow than controls
Schwepcke et al. (49)	SDF	Preterm neonates: postnatal hypertension vs. controls	10/11	Cutaneous (upper inner arm)	Preterm neonates with hypotension showed higher FVD in the first 6 h after birth; at 12 h after birth both blood pressure and FVD did not differ between groups
Tytgat et al. (12)	SDF	Neonates undergoing laparoscopic surgery for hypertrophic pyloric stenosis	12	Buccal and sublingual mucosa	Buccal FVD did not differ before and after surgery. Sublingual blood vessel diameters increased during CO_2_ insufflation and decreased after CO_2_ exsufflation
Ergenekon et al. (50)	SDF	Term neonates with HIE: TH vs. controls	7/7	Cutaneous (axilla)	Patients showed lower MFI and more vessels with sluggish flow than controls. After TH parameters recovered to values of controls
Buijs et al. (6)	SDF	Term neonates with CDH: catecholamines vs. controls	28/28	Buccal mucosa	Catecholamines improved the macrocirculation, but did not alter the microcirculation; impaired microcirculation was predictive of outcome
Van den Berg et al. (35)	SDF	Healthy term neonates	28	Cutaneous (upper inner arm)/buccal mucosa	Application SDF imaging; reproducibility of buccal PVD with SDF imaging was confirmed, cutaneous PVD showed poor reproducibility
Van Elteren et al. (17)	SDF/IDF	Healthy preterm neonates	20	Cutaneous (upper inner arm)	IDF imaging showed higher TVD and lower PPV values than SDF imaging because of higher image quality
Van Elteren et al. (51)	IDF	Healthy preterm vs. term neonates	60/33	Cutaneous (upper inner arm)	TVD decreased in first month of life in both groups; TVD was higher in preterm than in term neonates
Gassmann et al. (52)	IDF	Healthy term neonates: born at high altitude vs. born at sea level	53/33	Cutaneous (upper inner arm)	TVD was higher in neonates born at high altitude (lower SpO_2_ levels) than in neonates born at sea level
Wright et al. (36)	SDF	Healthy term neonates	42	Cutaneous (ear conch)	Application SDF imaging; reporting of reference values for microcirculatory parameters for ear conch
Kulali et al. (53)	SDF	Healthy term neonates: vaginal delivery vs. cesarean section	12/25	Cutaneous (axilla)	Vaginal delivery group showed more vessels with hyperdynamic flow than cesarean section group; other parameters did not differ between groups
Puchwein-Schwepcke et al. (54)	SDF	Term neonates: infection treated with antibiotics vs. controls	13/95	Cutaneous (ear conch)	Infection group showed lower FVD and higher proportion of hyperdynamic flow than control group; hyperdynamic flow was associated with 5-fold increased risk for infection
Puchwein-Schwepcke et al. (55)	SDF	Preterm neonates with extreme LBW: hypercapnia vs. controls (sub-analysis RCT)	6/6	Cutaneous (upper inner arm)	Hypercapnia group showed lower FVD and relatively fewer small vessels than controls

BP, blood pressure; CDH, congenital diaphragmatic hernia; ECMO, extracorporeal membrane oxygenation; etCO_2_, end tidal carbon dioxide; FVD, functional vascular density; GA, gestational age; Hb, hemoglobin; HIE, hypoxic ischemic encephalopathy; HR, heart rate; Ht, hematocrit; HVM, handheld vital microscopy; IDF, incident dark field illumination; LBW, low birth weight; OPS, orthogonal polarization spectral; PDA, persistent ductus arteriosus; PPV, perfused vessel density (%); RBC, red blood cell; SDF, sidestream dark field; TH, therapeutic hypothermia; TVD, total vessel density; VA ECMO, veno-arterial extracorporeal membrane oxygenation; VD, vessel diameter.

The authors apologize for this error and state that this does not change the scientific conclusions of the article in any way. The original article has been updated.

